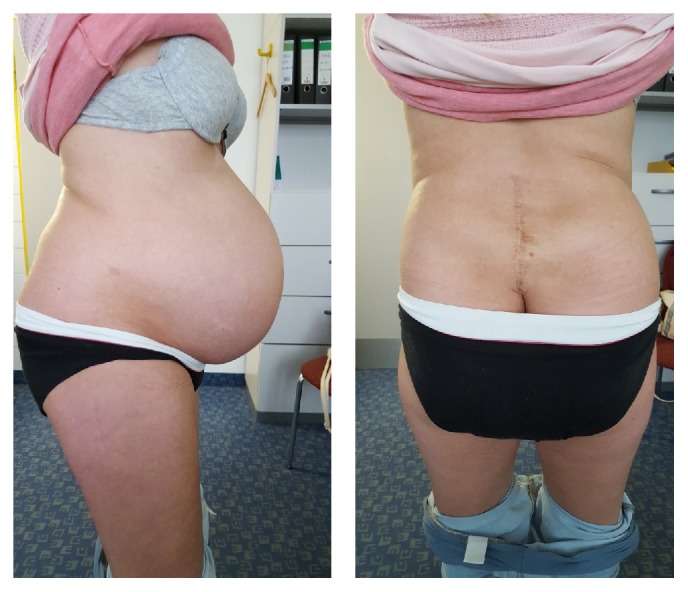# Successful Spontaneous Pregnancy after Treatment for Ewing Sarcoma including Sacrectomy

**DOI:** 10.1155/2018/2484036

**Published:** 2018-04-30

**Authors:** T. Hockertz, M. Velickovic

**Affiliations:** Department of Orthopedic Surgery, Sports Traumatology and Trauma Surgery, Städtisches Klinikum Wolfenbüttel, Alter Weg 80, 38302 Wolfenbüttel, Germany

## Abstract

Ewing sarcomas are highly malignant bone tumors and usually occur in childhood. Radiation therapy, chemotherapy, and surgical methods increase the survival rate of the affected patient, but infertility and reduced reproductive capacity are common late effects of pediatric cancer treatment.

## 1. Introduction

We report on the pregnancy of a patient with Ewing sarcoma treated at the age of 18 with chemo- and radiotherapy as well as total sacrectomy. In the course of time, a “neosacrum” developed and the patient gave birth to a boy at the age of 29. The course of pregnancy was uneventful. This was the second pregnancy of this patient. At the time of pregnancy, the patient was 29 years old.

## 2. Case Presentation

**Figure 1:**
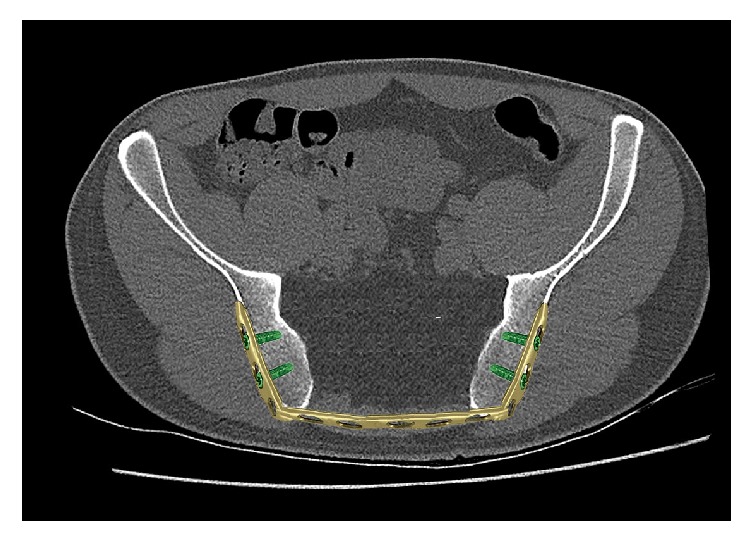
Schematic representation of the sacrectomy.

**Figure 2:**
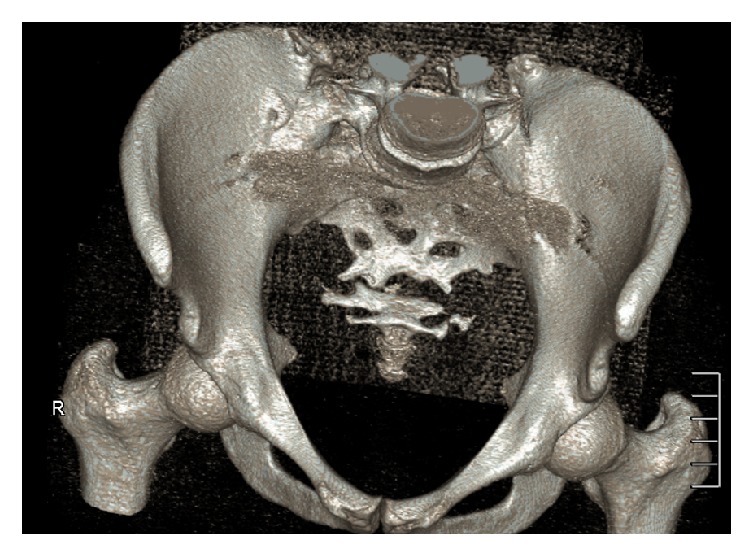
CT reconstruction of the pelvis showing the “neosacrum.”

**Figure 3:**
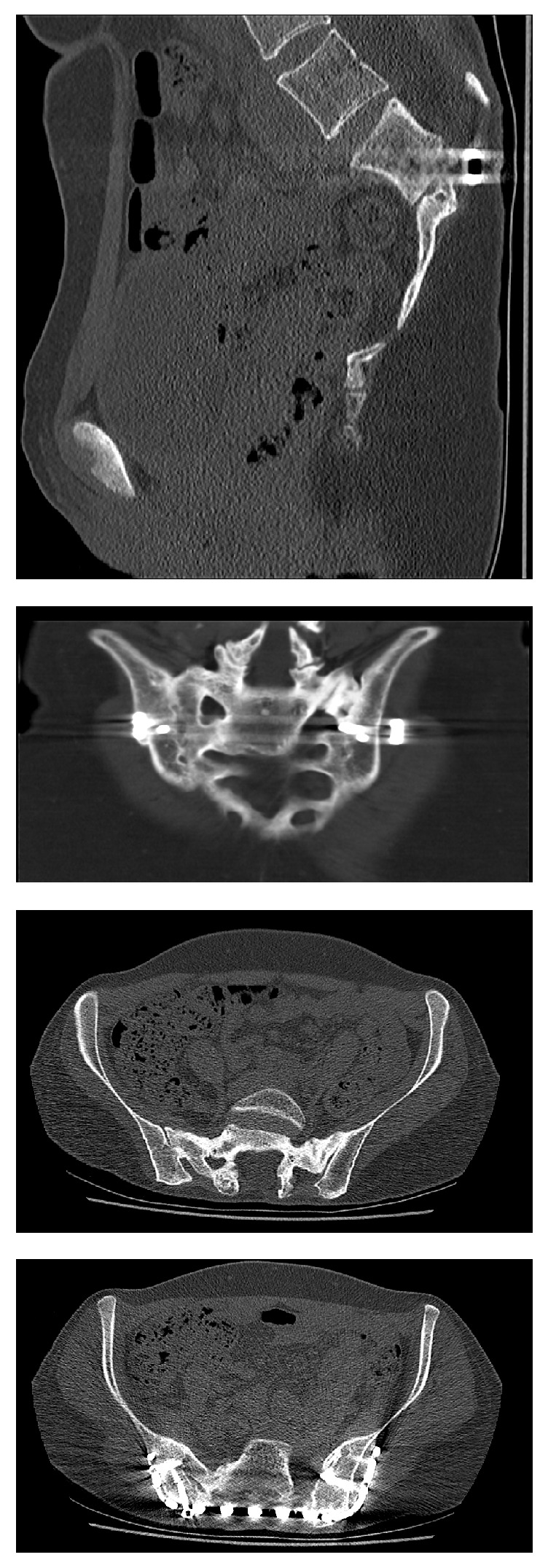
Selection of CT slices of the pelvis (CT and X-ray of the lower spine and pelvis were done in another hospital in 2015 due to lower back pain).

The patient was 18 years old at the time Ewing sarcoma of the sacrum was diagnosed. At the time when the patient consulted for the first time a doctor, she was already suffering from progressive pain in the lumbosacral region radiating into both legs with saddle block anesthesia, foot flexor paresis, and episodes of bladder and stool incontinence for four months. An initial MRI of the lower spine suggested a tethered cord syndrome and the patient was transferred and operated on in a neurosurgical clinic, but the patient did not benefit from the surgery. In an additional MRI of the pelvis, a large tumor was found. The tumor was partly intra-, partly extraosseous and involved the entire right sacrum wing and extended into the small pelvis and the dorsal pelvic soft parts. The histopathological examination of the sample biopsy confirmed Ewing sarcoma. Staging showed no evidence of metastasis of the tumor. The stage was documented with T3 N0 M0. We treated the patient with a combination of neoadjuvant chemotherapy, radiation therapy, and sacrectomy. In the first preoperative phase, the patient received 3-day blocks of polychemotherapy consisting of vincristine, etoposide, doxorubicin, and ifosfamide. This was performed preoperatively six times at intervals of 3 weeks. The tumor showed a marked reduction in size from 9 cm × 6.5 cm × 12 cm before the start of the therapy and 8 cm × 5,5 cm × 10,5 cm after 6 chemotherapy blocks (data in each case in width × depth × height). Total sacrectomy followed. In a first step, the position of the lumbar spine was secured with a lumbopelvic fixation from the ileum to the third lumbar vertebral body. The tumor could not be removed en bloc, so we performed an intralesional tumor resection. Before complete dissection of the sacrum was done, we placed an angular-stable ilioiliacal LCP plate sacrum in order to ensure an anatomical position of the pelvis (Figure 1). Postoperatively, we continued chemotherapy as well as radiation therapy due to the intralesional tumor resection. The radiation dose was 45 Gy. The follow-up period was 12 years. The radiological follow-up examinations over the course of time documented a nearly complete sacral osteoneogenesis including the neuroforamina with a stable fusion of the new sacrum with the posterior pelvic ring (Figures 2 and 3). The clinical outcome was very good, and the gait was almost normal; no crutches were needed anymore. Neurologically, an incomplete paraplegia remained, with preserved motor function and sensitivity below the spinal cord injury site L5/S1 with a clinically nonrelevant peroneal paresis on the right, discrete paresthesia on the right lateral thigh and distal lower limbs, and a neurogenic bladder dysfunction.

## 3. Pregnancy

Contraception was done with birth control pills. The patient wanted to conceive so this medication was stopped. Three months later, the patient was pregnant at the age of 28 but lost the child in the 8th week of pregnancy. Curettage was necessary. The second pregnancy at the age of 29 was successful and she gave birth to a boy of 3460 g and 50 cm through a cesarean section at the 37 weeks' gestation by a community obstetrician. There were no birth complications (Figure 5).

## 4. Discussion

Ewing sarcoma is a highly malignant bone tumor which rapidly metastasizes at an early stage. Treatment options include radiation, chemotherapy, and operation. The combination of chemotherapy with both surgery and radiotherapy increases the survival rate by 15–20% as compared to chemotherapy with either local therapy alone [1, 2]. Prognosis has been improved in recent years. In the 1960s, the 2-year survival rate was only 21%. Due to modern treatments, long-term survival up to 70% to 80% can be reached among patients without metastases [3, 4]. The aim of treatment is to cure the patient with the least cost in terms of long-term morbidity.

A major fear among female survivors is the long-term effects of cancer therapy especially infertility. Family planning is for most people an essential part of life quality. In general, cancer survivors are less likely to become pregnant when compared to their siblings [5–7].

Ewing sarcoma occurs mainly in childhood. Strategies for preserving fertility are limited and should be planned before starting tumor therapy and discussed with the patient and family. However, this is often not possible due to the urge to start with the therapy. In general, we recommend an interdisciplinary team including a gynecologist in order to choose the best way to preserve fertility. Even in prepubertal girls, the physician should keep the possible wish to have children later on in mind. In our case, the patient was 18 years old when Ewing's sarcoma was diagnosed. There was no gynecologist involved and no pretreatment to preserve fertility was done.

According to Lee et al., there are several well established methods to preserve fertility, including gonadal shielding during radiotherapy, trachelectomy, and ovarian transposition. Methods like embryo cryopreservation are only useful in established stable partnership which is unlikely at the patient's age. Other methods like oocyte cryopreservation, ovarian cryopreservation and transplantation, and ovarian suppression with GnRH analogs or antagonists are experimental. But Lee et al. also highlighted that ovarian tissue cryopreservation and reimplantation is a main option to preserve fertility of cancer patients who need an immediate start of the cancer treatment. For patients in the prepubertal age, freezing is the only option to preserve fertility [8]. Up to now, a total of 17 babies from 12 patients have been born worldwide from ovarian tissue cryopreservation and reimplantation [8–10]. Three of them suffered from Ewing's sarcoma.

Treatment with multiagent chemotherapy, radiotherapy, and surgical procedures such as sacrectomy is associated with significant late effects and reduces the chance of pregnancy [11].

Radiation therapy leads to decreased fertility depending on the site irradiated. Ewing's sarcoma most commonly affects the femur and the pelvis in 20% of cases, so the female reproductive system is often affected. The younger the patient at the time of radiation, the greater the probability of infertility. The risk of pregnancy complications such as miscarriage, preterm delivery, and perinatal death is higher. There is also a higher risk of low birth weight less than 2500 g in infants born to patients treated with pelvic irradiation [12]. There is no known radiation dose threshold for uterine damage, but abdominal radiation with 20–30 Gy in childhood already leads to ovarian failure [13]. According to Teh et al., no successful pregnancy been reported after a direct radiation dose (>45 Gy) to the whole pelvis [14]. Our patient received a radiation dose of 45 Gy with no protective measures like ovarian transposition.

Haerr and Pratt recommend early chemotherapy in the course of many malignant sarcomas, despite pregnancy, to prevent the occurrence of metastases [15]. It is known that busulfan and high doses of lomustine are significantly associated with reduced pregnancy in female survivors of childhood cancer not exposed to pelvic or cranial radiotherapy. According to the same authors, chemotherapy-specific effects on pregnancy were generally few in patients without radiotherapy to the pelvis [16].

There is only one case in the literature which is similar to our case.

Kakogawa et al. reported a case of successful pregnancy in a patient who underwent sacrectomy combined with multiagent chemotherapy and radiotherapy for Ewing's sarcoma. The patient was diagnosed with Ewing's sarcoma of the sacrum at the age of 16. But in this case, pretreatment in order to protect the reproductive system was carried out, the ovaries were transposed, the uterus was shielded, and a gonadotropin-releasing hormone agonist was used during treatment to protect the ovarian function. The patient spontaneously conceived at the age of 27 [4, 17]. On the other hand, a spontaneous return of ovarian function and conception years after gonadal chemotherapy with evidence of ovarian failure is possible [18].

At the time the patient arrived at our hospital, she already suffered from severe neurologic symptoms. Due to the urgency to start with the therapy, no precautions were taken to maintain fertility.

**Figure 4:**
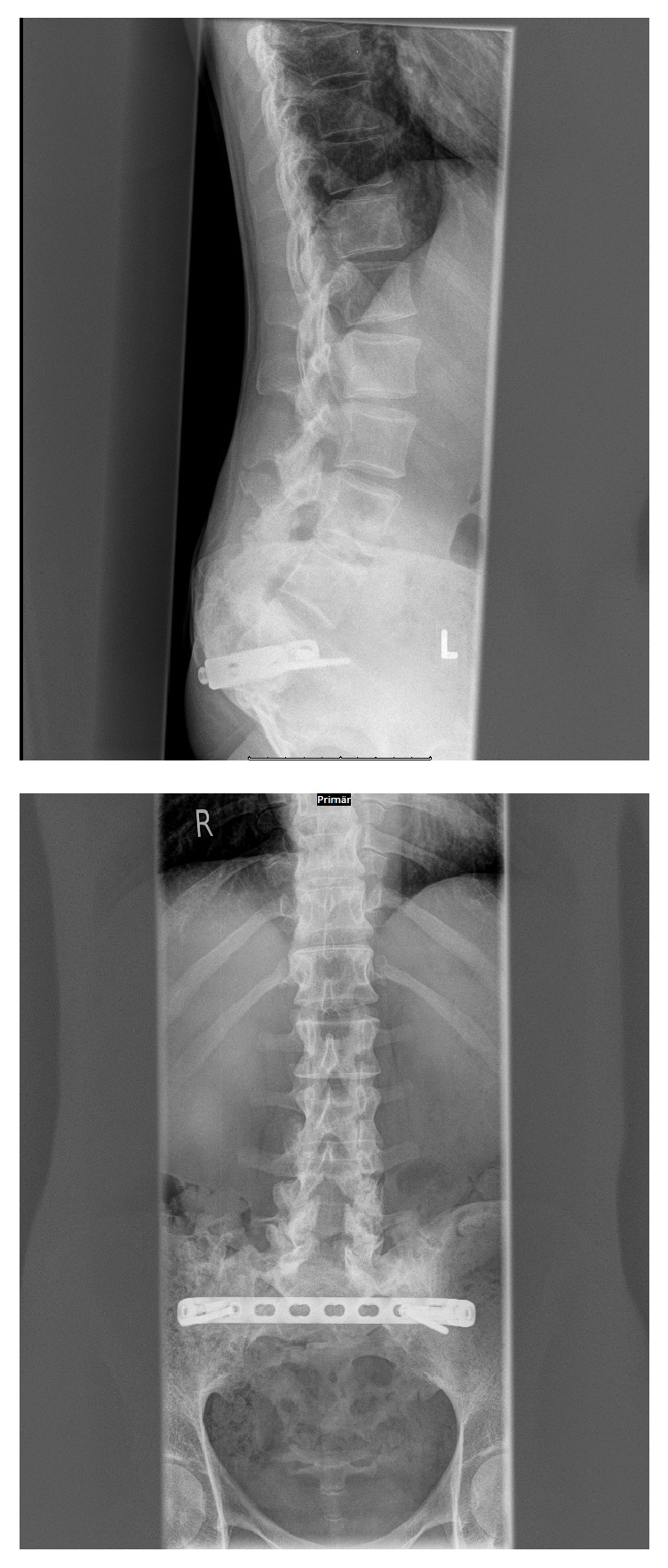
X-ray of the lower spine with ilioiliacal plate.

This is the 6th reported case of pregnancy in a patient with Ewing's sarcoma [4, 19–21]. All women became naturally pregnant. The pregnancy course was in 4 cases uneventful; in one case only, an inadequate descent of the fetal head occurred due to pelvic distortion. In 3 cases, a caesarian section was done. In the other cases, the way of delivery is unknown. We also recommend a caesarian section. Biomechanically, the ilioiliacal pelvic plate acts as an internal fixator which prevents widening of the posterior pelvic ring at birth (Figure 4). In the case of a vaginal birth, the baby could be stuck in the birth canal or the pelvic ring may burst open. Both would put the mother and the child in unnecessary danger. Neurologically, an incomplete paraplegia remained, with preserved motor function and sensitivity below the spinal cord injury site L5/S1 with a clinically nonrelevant peroneal paresis on the right, discrete paresthesia on the right lateral thigh and distal lower limbs, and a neurogenic bladder dysfunction. Neurological deficits are not an absolute indication for cesarean delivery but caesarian section is recommended to reduce the risk for the baby.

Successful and uneventful pregnancy after sacrectomy combined with chemotherapy and radiotherapy can be achieved even without pretreatment for fertility preservation.

## Figures and Tables

**Figure 5:**